# Determination of bisphenol a migration from food packaging by dispersive liquid-liquid microextraction

**DOI:** 10.1016/j.mex.2021.101415

**Published:** 2021-06-15

**Authors:** Mostafa Mahdavianpour, Narges Chamkouri, Hossein Chamkouri, Zahra Kolivand, Najaf Noorizadeh, Seyed Mohammad Ali Malaekeh, Soraya Karami

**Affiliations:** a Abadan University of Medical Sciences, Abadan, Iran; b Faculty of Chemical and Petroleum Engineering, University of Tabriz, Tabriz, Iran

**Keywords:** Dispersive liquid-liquid microextraction, Packaged foods, Bisphenol A

## Abstract

In the current work, a rapid and simple dispersive liquid-liquid microextraction method (DLLME) was used to determine Bisphenol A (BPA). High performance liquid chromatography with the photodiode-array detector (HPLC-DAD) coupled DLLME method was employed to analyze BPA in food samples packaged including cans, paper boxes, and glass jars. The calibration curve was obtained to be in the linear range 0.009–25 ngg^−1^ with a correlation coefficient of R2 = 0.9981. The mean relative standard deviations (RSDs) was of 5.2% (*n* = 3). The limit of detection (LOD) and the limit of quantification (LOQ) of the method were obtained to be 0.001 ngg^−1^ and 0.08 ng.g^−1^, respectively.

In sum, this method presents:

• A rapid, simple and efficient modified DLLME method was used to measure BPA in packaged foods.
• The advantages of this method were low detection limit, fast preparation, and high BPA recovery.
• The DLLME-HPLC method consists of low detection limit and high recoveries to determine BPA in samples.
• The results indicated that DLLME –HPLC-DAD was an applied method to measure BPA in food samples.

## Graphical abstract

**Figure:**
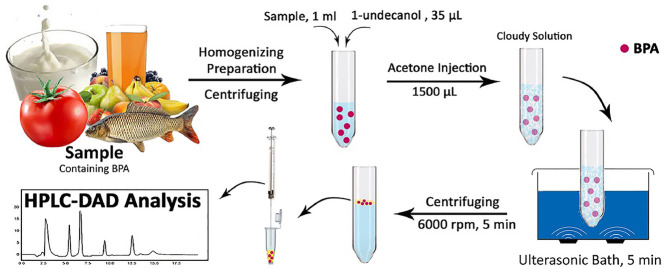

Specifications table

**Table:** 

Subject Area:	Food science
More specific subject area:	Chemistry of food products
Method name:	Measurement of Bisphenol A by Dispersive Liquid-Liquid Microextraction Coupled HPLC-DAD in Packaged Foods
Name and reference of original method:	Sungur Ş, Köroğlu M, Özkan A. Determinatıon of bisphenol a migrating from canned food and beverages in markets. Food chemistry. 2014; 142:87-91. Liu X, Ji Y, Zhang H, Liu M. Elimination of matrix effects in the determination of bisphenol A in milk by solid-phase microextraction–high-performance liquid chromatography. Food additives and contaminants. 2008; 25(6):772–8.
Resource availability:	None

## Method details

The widespread use of chemicals in the food industry can be a health danger for humans and animals (especially pets) [1,2]. Bisphenol A (BPA) as a common monomer is used to produce epoxy resins and polycarbonates [1]. BPA migration from packaging materials was identified in many packaged foods such as vegetables, soft drinks [1,2]. Chemical compounds, such as Bisphenol A (BPA), can be carcinogenic and damage the liver, bones, nerves, and also endocrine system [2,3]. Important properties of BPA comprise moderate water solubility, low vapor pressure, low volatility, and its solid state at ambient temperature [4]. So, because of differences between food industries around the world, it is necessary to monitor the presence of BPA in different packaged foods and different food brands [1], [2], [3], [4].

Several pre-concentration methods, such as liquid–liquid extraction (LLE), solid-phase extraction (SPE), and dispersive liquid–liquid microextraction (DLLME), have been developed to determine BPA [4], [5], [6], [7], [8]. DLLME is similar to LLE with an extraction solvent in the microliter range [4], [5]. DLLME is a novel and powerful pre-concentration method with a small volume of the disperser solvent and the extraction solvent. In the DLLME method, an appropriate mixture solvent is rapidly injected into the sample, and then, the mixture is gently shaken, and a cloudy solution containing fine droplets is dispersed in the sample solution [4]. The advantages of the DLLME method are short extraction time, fast preparation, low cost, low sample cleanup, and high enrichment factor. The main purpose of this work was to develop a simple, accurate, and sensitive method to measure BPA in different food samples, using HPLC-DAD after pre-concentration by the DLLME procedure.

## Chemicals and reagents

BPA, acetonitrile (ACN) and methanol were purchased from Sigma–Aldrich (HPLC, gradient grade, USA, purity≥99%). Also, all the other chemicals and reagents were obtained from Sigma–Aldrich (analytical grade, purity ≥99%). High purity ultrapure water was supplied from Millipore, Milli-Q (Bedford, MA, USA).

## Instrumentation

High-performance liquid chromatography equipped with photo Diode-Array detector (Knauer, DAD 2100, wavelength range190–700 nm, and accuracy ± 1 nm) were used for chromatographic determination of BPA in the food samples. HPLC-DAD was equipped with micro-vacuum degasser and 0.1 µL auto-sampler. The Ez Chrom Elite software was used to calculate peak areas. Chromatographic separation was employed on C18 column (5 m, 250 × 46 cm, 5 µm particle size) with pre-column 50 × 8 mm. The binary mobile phase, including water and acetonitrile (55: 45% v/v), was pumped at the column oven as solvent. The pH was measured with Metrohm pH meter (model 827) supplied with a glass-combined electrode. A Hettich Zentrifugen (EBA20, Tuttlingen, Germany) device was used for centrifugations.

## Chromatographic conditions

A simple HPLC-DAD analysis method was used to measure BPA in the prepared samples. Analytical parameters affecting HPLC signals, including the ratios of the solvents and the column oven temperature, were studied and optimized. In the HPLC method, the mobile phase is in an isocratic elution mode. The mobile phase including water and acetonitrile (55:45% v/v) was pumped at the column oven at the temperature range of 20 °C – 45 °C with a flow-rate of 0.5 mL min^−1^. According to the results, the temperature of 25 °C was chosen as the optimum value in the subsequent analyses. In addition, 20 μL of the sample extract was injected automatically. Furthermore, BPA was detected at a wavelength of 254 nm, and the HPLC calibration curve was plotted in the range of 0.009–25 ngg^−1^ BPA with R^2^> 0.99.

## Standard preparation

In this study, a standard stock solution containing 100 mgmL^−1^ of BPA was prepared in methanol. The working solutions were prepared by appropriate dilution of the standard stock solution in methanol. To prepare a standard stock solution, 5 g of BPA was dissolved in 50 mL methanol. Six-point working standard solutions containing BPA were prepared at concentrations of 0.009, 1, 5, 10, 25, and 50 mgmL^−1^. All the stock solution and working standards were stored at 4 °C and brought to room temperature before use. Solvents, standard solutions, and samples used to perform the HPLC application were filtered through a 0.22 µm nylon filter (Varian, USA).

## Sampling procedure

A total of 24 different packaged foods, including canned foods, foods kept in paper boxes, and food sold in glass jars, were randomly supplied from local supermarkets in Abadan, southwest of Iran. The canned foods comprised of 3 brands of tomato, apple fruit, fish, and powdered milk. The juices and milk represented foods in paper boxes (3 brands of each foods). Also, two kinds of foods, tomato and apple, were taken as samples of foods sold in glass jars. All samples were stored at room temperature (± 25 °C) and kept unpacked before the analysis. After opening the foods packages, the total contents of each packing were homogenized, and an aliquot sample was taken for the required analyses.

## Preparation of food samples

For preparing tomato and fruit samples, 5.0 g of homogenized contents of packages were added into a screw-capped glass vial, and 3 mL of ultra-pure water was also added. Prepared sample in vial was mixed, using a vortex for 4–5 min, and then centrifuged at 4000 × g for 2 min. After centrifugation, 5 mL of acetonitrile, 2.0 g of anhydrous MgSO_4_ and, 0.5 g of NaCl were added to the mixture. The new mixture was shaken for 15 min and centrifuged again at 4000 × g for 2 min. The final mixture was collected in a volumetric flask, and its volume increased to 50.0 mL by adding ultra-pure water.

For canned fish samples, 0.1 g of each fish tuna was added to 5.0 mL of acetonitrile in a screw-capped glass vial. The mixture was homogenized by an ultrasonic bath for 30 min, and then centrifuged at 1500 × *g* for 15 min. The extracted phase was mixed with 15 mL of *n-*hexane and collected in a volumetric flask and its volume increased to 50 mL using ultra-pure water. The pH value of samples was adjusted to neutral range with sodium hydroxide (1 M).

For preparation of powdered milk samples, 0.5 g of homogenized powdered milk with 2.5 mL of ethanol (50%, w/v) was added to a screw-capped glass vial. The sample was degassed in ultrasonic bath for 3 min, and then centrifuged at 5000 × g for 35 min. Finally, centrifugation supernatant was filtered and poured in a volumetric flask, and its volume was increased to 50 mL by adding ultra-pure water. Finally, the samples were adjusted to pH 7.0 with sodium hydroxide.

For juices and milk samples, 10.0 mL of homogenized liquid was added to a glass vial. The pH of samples was adjusted to 7.6 using buffer phosphate. The sample was homogenized in an ultrasonic bath for 3 min, and finally was filtered.

## DLLME-HPLC-DAD method

**Fig. 1:**
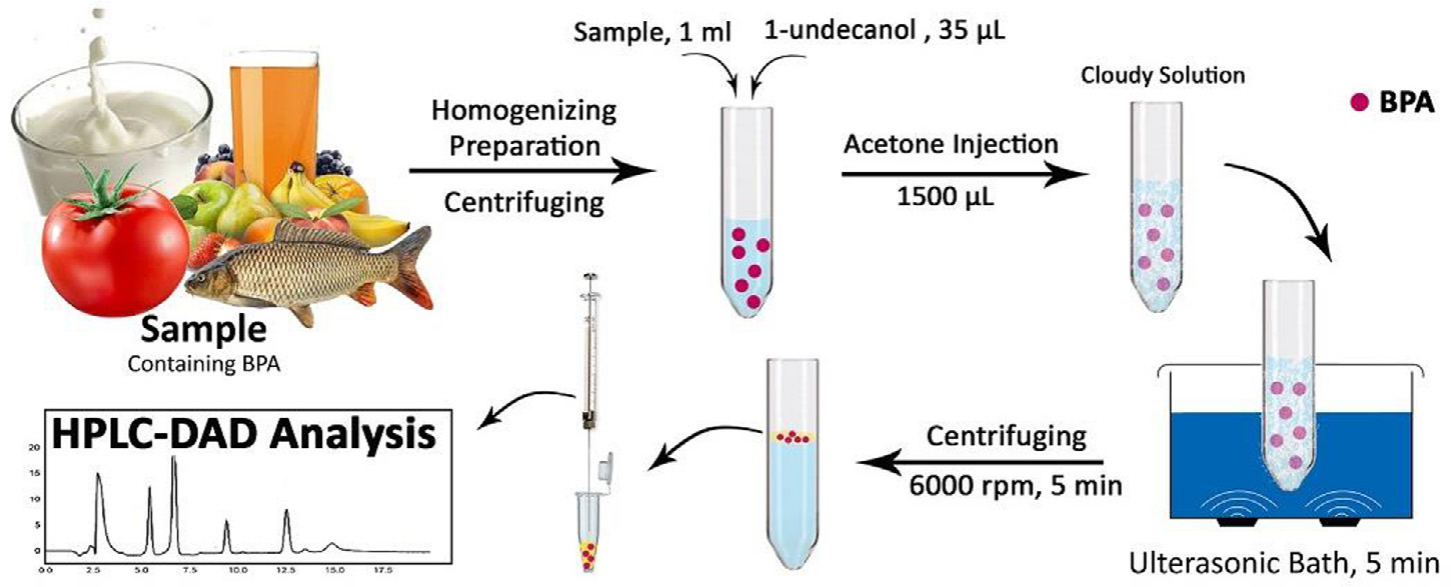
Schematic representation of the sample preparation of DLLME-HPLC-DAD.

In this method, influence parameters on BPA extraction including, ultrasonic time, volume and type of solvents, and salt effect as ionic strength optimized. Fig. 1 shows the steps of the DLLME method. Sample preparation of procedure include: (a) Injection of 1-undecanol as an extracting solvent (35 µL) and of acetone as a dispersive solvent (1500 μL). (b) Mixing the ultrasonic water bath for 5 min. (c) Mixture centrifuged at 6000 rpm for 5.0 min (d) Injection into HPLC-DAD. In fact, DLLME method was performed without any salt addition.

## Optimization of DLLME factors for determination BPA in canned foods

**Fig. 2:**
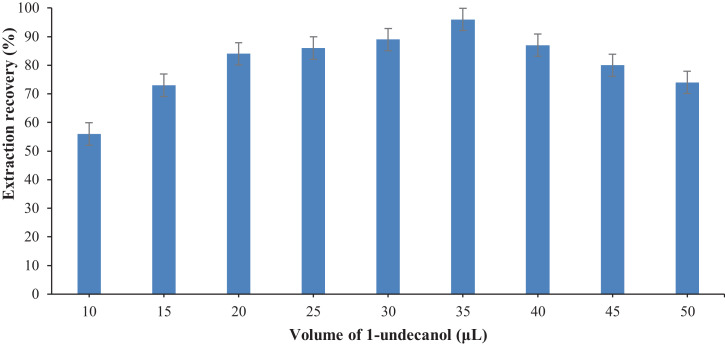
Optimization the volume of extraction solvent.

Optimization the type and volume of the extraction solvent is necessary in this method. The important properties of the extraction solvent are low volatility and low solubility in water for the formation of a cloudy phase in water. For this purpose, several solvents such as 1-decanol, 1-dodecanol, carbon tetrachloride, and 1-undecanol were tested as extraction agent. The obtained results showed that 1-undecanol has the highest extraction recovery in comparison with the other investigated solvents. Therefore, 1-undecanol was selected as the best extraction solvent. Also, for solvent volume optimization, different volumes of 1-undecanol (10–50 μL) were studied in the BPA extraction and recovery. The results of BPA recovery are shown in Fig. 2. The highest BPA recovery was obtained for 35 μL of extraction solvent.

**Fig. 3:**
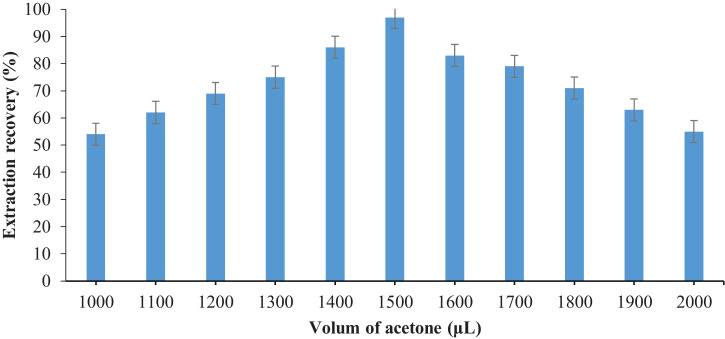
The effect of the volume of dispersive solvent on the recovery of BPA.

To investigate the effect of dispersive solvents on BPA recovery, different solvents, including acetone, acetonitrile, methanol, and ethanol, in the range of 1000–2000 μL were studied. According to Fig. 3, the highest BPA recovery for dispersive solvents obtained at 1500 μL of acetone.

Extraction time may affect the BPA recovery of the DLLME method. Extraction time is the time interval between injection of mixture of dispersive solvent and extraction solvent and starting of centrifugation. Several extraction times, in the range of 0–10 min, were studied with the optimum concentrations of extraction and dispersive solvents. The samples mixed in an ultrasonic water bath for 5 min.

Another probable effective factor in DLLME method is the effect of ionic strength on BPA extraction recovery. For this purpose, sodium chloride, at concentrations of 0–5% w/v, was used. The highest extraction recovery of BPA was obtained in the absence of sodium chloride. This phenomenon was because of the dispersive solvent solubility in water in presence of sodium chloride. Finally, mixture samples were centrifuged at 6000 rpm for 5.0 min.

## Method validation DLLME-HPLC-DAD for determination BPA

In this work, experimental procedure was validated for relative standard deviation (RSD), the limit of detection (LOD), limit of quantification (LOQ), linearity, correlation coefficient, recoveries, and enrichment factor (EF). The results of method validation are shown in Table 1. The LOD and LOQ for BPA were calculated at signal-to noise (S/N) ratio of 3 and 10, respectively. The LOD and LOQ were obtained to be 0.01 ngg^−1^ and 0.08 ng.g^−1^, respectively. The linearity of BPA was obtained in the range of 0.009–25 ngg^−1^ with the correlation coefficient of R^2^=0.9981. The precision of the method was investigated in term of relative standard deviations (RSDs), and was less than 5.2%. According (Eq.(1)), the EF defined as the ratio between BPA concentrations of in sediment extraction solvent (C_sed_) and the concentration of BPA in the real sample (C_0_). EF was obtained to be 1980 at the concentration level of 2 ngg^−1^.

(1) $$EF=\frac{C_{sed}}{C_{0}}$$

**Table 1:** Method of evaluation of method for determination of BPA.

Parameter	Value
Limit of detection (ngg^−1^, *n* = 3)	0.001
limit of quantification(ngg^−1^, *n* = 3)	0.08
Repeatability, RSD (%, *n* = 3)	5.2
Linear range (ngg^−1^)	0.009–25
Enrichment Factor	1980
R^2^	0.998

Repeatability of DLLME method was obtained at BPA concentration of 2 ngg^−1^. The data of the method evaluation revealed that DLLME-HPLC method was sensitive for determination of BPA.

## Determination BPA concentration and recovery in different food samples

The proposed DLLME-HPLC-DAD was used to measure BPA concentration in different food samples. Recovery is obtained according to Eq. (2), in which C_found_ is the total concentration of BPA found after addition of standard, C_real_ is the real concentration of BPA, and C_add_is the concentration of standard sample was spiked into the real sample.

(2) $$Recovery=\frac{C_{found}-C_{real}}{C_{add}}\times 100$$

**Fig. 4:**
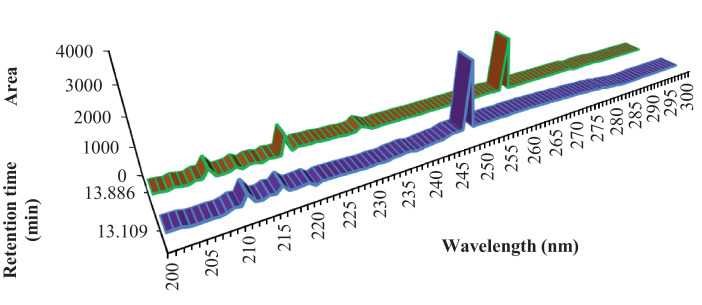
Separation of BPA in juices (blue) and fruits (green). (For interpretation of the references to color in this figure legend, the reader is referred to the web version of this article.).

BPA concentration and recovery of different food samples are presented in Table 2. BPA chromatogram of tomatoes (a), juices (b), and fruits (c) are shown in Fig. 4, respectively. As can be seen, the retention time of BPA was around 13 min, at BPA concentration of 10 ngg^−1^.

**Table 2:** BPA concentration and recovery of different food samples.  ND; not detected.

Packaging	Food type	Brand	Total BPA concentration (ng/g)	Added BPA (ng/g)	Measured BPA (ng/g)	Recovery (%)
Canned foods	Tomato	A	7.8 ± 0.43	5	4.6	92
		B	20.1 ± 1.9	5	4.4	88
		C	15.6 ± 0.13	5	5.02	100.4
	Fruit	D	18.04±1.07	5	4.8	96
		E	19.5 ± 0.02	5	4.5	90
		F	14.01±0.08	5	3.9	79
	Fish	G	10.5 ± 2.2	10	9.4	94
		H	8.6 ± 0.03	10	9.3	93
		I	15.9 ± 4.1	10	10.06	100.6
	Powdered milk	J	n.d	2	2.02	101
		K	4.88±0.6	2	1.53	76.5
		L	4.01±0.09	2	1.88	94
Paper box	Liquid milk	P	n.d	0.5	0.45	90
		Q	1.5 ± 0.04	0.5	0.52	104
		R	1.3 ± 0.08	0.5	0.38	76
	Juice	M	1.2 ± 0.5	2	1.9	95
		N	1. 2 ± 0.9	2	2.07	103
		O	Nd	2	2.02	101
Glass Jar	Tomato	S	Nd	0.5	0.41	82
		T	1.6 ± 0.01	0.5	0.52	104
		U	0.08±0.07	0.5	0.49	98
	Fruit	V	1.1 ± 0.04	1	0.91	91
		W	ND	1	0.93	93
		X	0.7 ± 0.003	1	0.97	97
